# Supplementary material for: Efficacy and safety of Dachaihu Decoction for acute pancreatitis: Protocol for a systematic review and meta-analysis
Source: PLoS One. 2023 May 18;18(5):e0285661. doi: 10.1371/journal.pone.0285661 (PMC10194870; doi:10.1371/journal.pone.0285661)
Supplement: S1 Appendix — (PDF) [file pone.0285661.s002.pdf]

The effectiveness and safety of Dachaihu Decoction for acute pancreatitis: a systematic review and meta-analysis

Citation

Xiang Xiao, Xuanyu Wu, Qinwei Fu, Xiao Pang, Yuanyuan Li, Qinxiu Zhang. The effectiveness and safety of Dachaihu Decoction for acute pancreatitis: a systematic review and meta-analysis. PROSPERO 2021 CRD42021245735 Available from: https://www.crd.york.ac.uk/prospero/display_record.php?ID=CRD42021245735

Review question

Dachaihu Decoction, a traditional herbal formula, is widely administered as an acute pancreatitis treatment in China. However, there are no systematic review on the effectiveness and safety of such therapy for treating acute pancreatitis. The aim of this study is to evaluate the effectiveness and safety of Dachaihu Decoction as used in the treatment of AP.

Searches

The Cochrane Library, PubMed, Embase, Web of Science, ScienceDirect, the WHO International Clinical Trials Registry Platform, Chinese Clinical Trial Registry, Google Scholar and ClinicalTrials.gov, the China National Knowledge Infrastructure (CNKI) database, the Wanfang Database, the Chinese Science Journal Database (VIP), and the Chinese Biological Medicine Literature Service System (CBM) will be searched for eligible randomized controlled trials of Dachaihu Decoction for the treatment of acute pancreatitis from inception of the database to April 2021.

Search strategy

https://www.crd.york.ac.uk/PROSPEROFILES/245735_STRATEGY_20210328.pdf

Types of study to be included

Only randomized controlled trials (RCTs) of DCD for AP or publication status will be included.

Condition or domain being studied

Acute pancreatitis (AP), an inflammatory disorder of the pancreas, is the edema, hemorrhage, necrosis and self-digestion of the pancreas caused by multiple factors. Patients with AP can easily lead to intensive care unit admission and even death. Systemic inflammatory response syndrome (SIRS) and multiple organ dysfunction syndrome (MODS), which always lead to poor prognosis, are common because of the gastrointestinal disorder. AP is one of the most common acute abdominal disease in clinic, with prevalence estimated as about 40-45 of 100, 000 individuals per year in the US.In spite of the rapid development of modern medicine and the diagnosis, classification and management of the patients with AP, it is still a diseases of high mortality.Furthermore, the usage of antibiotics and surgical operation is still controversial.

Dachaihu Decoction (DCD) is a traditional Chinese medicine formula originated more than 1800 years ago, which was first described by a Chinese doctor called Zhongjing Zhang in "Treatise on Febrile Disease Caused by Cold (Shanghan Lun)". It consists of Bupleurum, Baikal Skullcap, Pinellia ternata, Chinese herbaceous peony, Chinese rhubarb, Fructus aurantii Immaturus, Zingiberis Rhizoma, Chinese date. DCD has a positive effect on AP and other diseases such as type 2 diabetes, fatty liver and so on.

Participants/population

Participants diagnosed with AP and treated with DCD in experimental group will be included. The restriction of age, gender and ethnicity of the enrolled subjects is not restricted.

Intervention(s), exposure(s)

The participants in experimental group were treated with DCD.

Comparator(s)/control

The participants in the control group were treated with pure western medicine, other traditional Chinese medicine, or other treatment without DCD.

Main outcome(s)

The primary outcomes include the overall response rate, mortality, surgical intervention rate. The overall response rate was calculated as: (cure number + effective number)/ total number * 100%. The mortality refers to intra-hospital mortality. The surgical intervention rate was calculated as: surgical number/ total number * 100%.

Measures of effect

Overall response rate, mortality and surgical intervention will be considered as dichotomous data. We will calculate risk ratio (RR) for the dichotomous data. P-values less than 0.05 ($p<0.05$) will be considered statistically significant.

Additional outcome(s)

The secondary outcomes include the recovery time of abdominal pain disappearing, and the recovery time of serum amylase, serum lipase, C-reactive protein, IL-8 counts, TNF-α counts become normal. And the incidence of various complications.

Measures of effect

The recovery time of abdominal pain disappearing, the recovery time of serum amylase counts, serum lipase counts, C-reactive protein counts, IL-8 counts and TNF-α counts become normal, and the incidence of various complications will be considered as continuous data. We will calculate the 95% confidence interval (CI) and mean difference (MD) for the continuous data. P-values less than 0.05 ($p<0.05$) will be considered statistically significant.

Data extraction (selection and coding)

Two reviewers (XX and WXY) will extract and manage data independently. They will check the result with each other. If disagreement occurred, a third author will judge it. Important information includes the name of the first author, year of republication, country of publication, average age of testee, average course of disease, classification of AP, sample size, interventions, frequency of intervention, dose, route of administration, outcomes (overall response rate, mortality, surgical intervention, the recovery time of abdominal pain disappearing, the recovery time of serum amylase, serum lipase, C-reactive protein, IL-8 counts, TNF-α counts become normal, and the incidence of various complications.), adverse reactions, and complication.

Risk of bias (quality) assessment

Two reviewers (XX and FQW) will assess the risk of bias in included studies by using the Cochrane “risk of bias” tool. The Cochrane “risk of bias” tool is available from the website: https://methods.cochrane.org/bias/resources/cochrane-risk-bias-tool. The risk of bias for each study will be graded as high, low, or unclear level. Any disagreement will be resolved through discussion.

Strategy for data synthesis [1 change]

The statistical analysis will be performed using RevMan 5.3 software. We will use the Cochran Q test and I^2 test to access the heterogeneity of included studies. If $I^2<50\%$ and $p>0.1$, we will use the fixed effect model, otherwise the subgroup analysis or sensitivity analysis will be conducted to find the source of heterogeneity and we will use the random effect model to merge the data. We will synthesise risk ratios for dichotomous data such as overall response rate, mortality and surgical intervention. And mean difference or standard mean difference for continuous data such as the recovery time of abdominal pain disappearing, the recovery time of serum amylase, serum lipase, C-reactive protein, IL-8 counts, TNF-α counts becoming normal.

Analysis of subgroups or subsets

Subgroup analysis is necessary if there is obvious heterogeneity. Subgroup analysis will be conducted based on the route, dose and frequency of administration, and age, gender, and classification of AP if necessary.

Contact details for further information

Xiang Xiao

724676369@qq.com

Organisational affiliation of the review

Hospital of Chengdu University of Traditional Chinese Medicine

Review team members and their organisational affiliations

Mr Xiang Xiao. Hospital of Chengdu University of Traditional Chinese Medicine

Miss Xuanyu Wu. Hospital of Chengdu University of Traditional Chinese Medicine

Mr Qinwei Fu. Hospital of Chengdu University of Traditional Chinese Medicine

Mr Xiao Pang. the Affiliated Hospital of Southwest Medical University

Miss Yuanyuan Li. the Affiliated Hospital of Southwest Medical University

Professor Qinxiu Zhang. Hospital of Chengdu University of Traditional Chinese Medicine

Type and method of review

Meta-analysis, Systematic review

Anticipated or actual start date

10 April 2021

Anticipated completion date

31 August 2022

Funding sources/sponsors

There is no funding for this study.

Conflicts of interest

Language

Chinese-HK, Chinese-Simplified, Chinese-Trad, English

Country

China

Stage of review

Review Ongoing

Subject index terms status

Subject indexing assigned by CRD

Subject index terms

Acute Disease; Drugs, Chinese Herbal; Humans; Pancreatitis; Phytotherapy

Date of registration in PROSPERO

23 April 2021

Date of first submission

28 March 2021

Stage of review at time of this submission [1 change]

**Stage**	**Started**	**Completed**
Preliminary searches	Yes	Yes
Piloting of the study selection process	Yes	No
Formal screening of search results against eligibility criteria	No	No
Data extraction	No	No
Risk of bias (quality) assessment	No	No
Data analysis	No	No

The record owner confirms that the information they have supplied for this submission is accurate and complete and they understand that deliberate provision of inaccurate information or omission of data may be construed as scientific misconduct.

The record owner confirms that they will update the status of the review when it is completed and will add publication details in due course.

Versions

23 April 2021

23 April 2021

10 May 2021
