# Supplementary material for: Efficacy and safety of Dachaihu Decoction for acute pancreatitis: Protocol for a systematic review and meta-analysis
Source: PLoS One. 2023 May 18;18(5):e0285661. doi: 10.1371/journal.pone.0285661 (PMC10194870; doi:10.1371/journal.pone.0285661)
Supplement: S2 Appendix — (PDF) [file pone.0285661.s003.pdf]

Reporting checklist for protocol of a systematic review and meta analysis.

		Reporting Item	Page Number
**Title**			
Identification	#1a	Identify the report as a protocol of a systematic review	page 1
Update	#1b	If the protocol is for an update of a previous systematic review, identify as such	n/a. This systematic review and meta-analysis is the first attempt to assess the published evidence of DCD for the management of AP.
**Registration**			
	#2	If registered, provide the name of the registry (such as PROSPERO) and registration number	page 3
**Authors**			
Contact	#3a	Provide name, institutional affiliation, e-mail address of all protocol authors; provide physical mailing address of corresponding author	page 1
Contribution	#3b	Describe contributions of protocol authors and identify the guarantor of the review	page 15
**Amendments**			
	#4	If the protocol represents an amendment of a previously completed or published protocol, identify as such and list changes; otherwise, state plan for documenting important protocol amendments	n/a. It is a new study that no one have researched.
**Support**			

Sources	#5a	Indicate sources of financial or other support for the review	page 14
Sponsor	#5b	Provide name for the review funder and / or sponsor	page 14
Role of sponsor or funder	#5c	Describe roles of funder(s), sponsor(s), and / or institution(s), if any, in developing the protocol	page 14
**Introduction**			
Rationale	#6	Describe the rationale for the review in the context of what is already known	page 4
Objectives	#7	Provide an explicit statement of the question(s) the review will address with reference to participants, interventions, comparators, and outcomes (PICO)	page 7
**Methods**			
Eligibility criteria	#8	Specify the study characteristics (such as PICO, study design, setting, time frame) and report characteristics (such as years considered, language, publication status) to be used as criteria for eligibility for the review	page 7
Information sources	#9	Describe all intended information sources (such as electronic databases, contact with study authors, trial registers or other grey literature sources) with planned dates of coverage	page 9
Search strategy	#10	Present draft of search strategy to be used for at least one electronic database, including planned limits, such that it could be repeated	page 9
Study records - data management	#11a	Describe the mechanism(s) that will be used to manage records and data throughout the review	page 10

Study records - selection process	#11b	State the process that will be used for selecting studies (such as two independent reviewers) through each phase of the review (that is, screening, eligibility and inclusion in meta-analysis)	page 10
Study records - data collection process	#11c	Describe planned method of extracting data from reports (such as piloting forms, done independently, in duplicate), any processes for obtaining and confirming data from investigators	page 10
Data items	#12	List and define all variables for which data will be sought (such as PICO items, funding sources), any pre-planned data assumptions and simplifications	page 11
Outcomes and prioritization	#13	List and define all outcomes for which data will be sought, including prioritization of main and additional outcomes, with rationale	page 8
Risk of bias in individual studies	#14	Describe anticipated methods for assessing risk of bias of individual studies, including whether this will be done at the outcome or study level, or both; state how this information will be used in data synthesis	page 11
Data synthesis	#15a	Describe criteria under which study data will be quantitatively synthesised	page 12
Data synthesis	#15b	If data are appropriate for quantitative synthesis, describe planned summary measures, methods of handling data and methods of combining data from studies, including any planned exploration of consistency (such as I2, Kendall's τ)	page 12
Data synthesis	#15c	Describe any proposed additional analyses (such as sensitivity or subgroup analyses, meta-regression)	page 12

Data synthesis	#15d	If quantitative synthesis is not appropriate, describe the type of summary planned	page 12
Meta-bias(es)	#16	Specify any planned assessment of meta-bias(es) (such as publication bias across studies, selective reporting within studies)	page 12
Confidence in cumulative evidence	#17	Describe how the strength of the body of evidence will be assessed (such as GRADE)	n/a

Notes:

- 1b: n/a. This systematic review and meta-analysis is the first attempt to assess the published evidence of DCD for the management of AP.
- 4: n/a.. It is a new study that no one have researched.
