# Supplementary material for: Efficacy and safety of Dachaihu Decoction for acute pancreatitis: Protocol for a systematic review and meta-analysis
Source: PLoS One. 2023 May 18;18(5):e0285661. doi: 10.1371/journal.pone.0285661 (PMC10194870; doi:10.1371/journal.pone.0285661)
Supplement: S3 Appendix — (PDF) [file pone.0285661.s004.pdf]

1、China National Knowledge Infrastructure (CNKI)（https://www.cnki.net/）

(主题=大柴胡汤 + 大柴胡) AND (主题=急性胰腺炎 + 胰腺炎 + AP + acute pancreatitis) AND (篇关摘=随机 + RCT)

Preliminary search date: January 25, 2023

Number of retrieved articles: 186

2、Wanfang Database（http://www.wanfangdata.com.cn/index.html）

主题:(大柴胡汤 or 大柴胡) and 主题:(胰腺炎 or 急性胰腺炎 or AP or acute pancreatitis) and 摘要:(随机 or RCT)

Preliminary search date: January 25, 2023

Number of retrieved articles: 256

3、VIP Database（http://lib.cqvip.com）

(((题名或关键词=大柴胡汤 OR 题名或关键词=大柴胡) AND (((题名或关键词=胰腺炎 OR 题名或关键词=急性胰腺炎) OR 题名或关键词=AP) OR 题名或关键词=acute pancreatitis)) AND (摘要=随机 OR 摘要=RCT))

Preliminary search date: January 25, 2023

Number of retrieved articles: 167

4、the Chinese Biological Medicine Literature Service System(http://www.sinomed.ac.cn/)

("随机"[摘要:智能] OR "随机对照"[摘要:智能] OR "rct"[摘要:智能]) AND ("胰腺炎"[标题:智能] OR "急性胰腺炎"[标题:智能] OR "AP"[标题:智能] OR "acute"[标题:智能] AND "pancreatitis"[标题:智能]) AND ("大柴胡"[标题:智能] OR "大柴胡汤"[标题:智能])

Preliminary search date: January 25, 2023

Number of retrieved articles: 103

5、PubMed（https://pubmed.ncbi.nlm.nih.gov/）

NO	Search terms	Results	Time
#1	Pancreatitis	126,956	02:47:23
#2	Acute Pancreatitis	19,263	02:47:28
#3	Acute Edematous Pancreatitis	19,263	02:47:38
#4	Peripancreatic Fat Necroses	19,263	02:47:43
#5	Pancreatitis, Acute Necrotizing	1,104	02:47:46
#6	Hemorrhagic Necrotic Pancreatitis	1,111	02:47:50
#7	Acute Necrotizing Pancreatitis	1,124	02:47:54
#8	OR/#1-#7	126,956	02:48:26
#9	Dachaihutang	2	02:48:36
#10	Da-Chai-Hu-Tang	20	02:48:56
#11	Dachaihu Decoction	8	02:49:34
#12	OR/#9-#11	28	02:49:51
#13	#8 AND #12	4	02:50:05
#14	randomized controlled trial	267,094	02:50:23
#15	controlled clinical trial	260,267	02:50:27
#16	randomized	546,869	02:50:31
#17	Randomly	162,046	02:50:36

#18	Trials	723,382	02:50:38
#19	RCT	18,237	02:50:44
#20	OR/#14-#19	994,797	02:51:03
#21	#13 AND #20	1	02:51:17

Preliminary search date: January 25, 2023

Number of retrieved articles: 1

6、Web of Science(http://www.isiknowledge.com)

TS = ("acute pancreatitis" OR "acute pancreatitides" OR "pancreatitis") AND TS = ("Dachaihutang" OR "Da-Chai-Hu-Tang" OR "Dachaihu Decoction")

Preliminary search date: January 25, 2023

Number of retrieved articles: 2

7、Cochrane Library (https://www-cochranelibrary-com-443--cams.naihes.cn/")

NO	Search terms	Results
#1	MeSH descriptor: [Pancreatitis] explode all trees	1449
#2	Acute Pancreatitis	2895
#3	#1 OR #2	3475
#4	Dachaihutang	2

#5	Dachaihu Tang	12
#6	Da-Chai-Hu-Tang	5
#7	Dachaihu Decoction	13
#8	#4 OR #5 OR #6 OR #7	25
#9	#3 AND #8	4

Preliminary search date: January 25, 2023

Number of retrieved articles: 4

8、Embase(https://www.embase.com)

NO	Search terms	Results
#1	pancreatitis:ti,ab,kw OR 'acute pancreatitis':ti,ab,kw OR 'acute pancreatitides':ti,ab,kw	100,012
#2	'acute pancreatitis'/exp	36,213
#3	#1 OR #2	105,534
#4	dachaihutang:ti,ab,kw OR 'dachaihu decoction':ti,ab,kw OR 'da chai hu tang':ti,ab,kw	38
#5	'daisaikoto'/exp	132
#6	#4 OR #5	148
#7	#3 AND #6	6

Preliminary search date: January 25, 2023

Number of retrieved articles: 6

9、Scopus (https://www.scopus.com)

((TITLE-ABS-KEY (da-chai-hu-tang) OR TITLE-ABS-KEY (dachaihutang) OR TITLE-ABS-KEY (dachaihu AND decoction))) AND ((TITLE-ABS-KEY (acute AND pancreatitis) OR TITLE-ABS-KEY (pancreatitis) OR TITLE-ABS-KEY (acute AND edematous AND pancreatitis) OR TITLE-ABS-KEY (acute AND necrotizing AND pancreatitis)))

Preliminary search date: February 1, 2023

Number of retrieved articles: 7

10、CINAHL (http://search.ebscohost.com)

(pancreatitis or acute pancreatitis) AND (dachaihutang or dachaihu decoction)

Preliminary search date: January 25, 2023

Number of retrieved articles: 0

11、OpenGrey (http://www.opengrey.eu)

(Pancreatitis OR Acute Pancreatitis OR Acute Edematous Pancreatitis OR Acute Necrotizing Pancreatitis) AND (Dachaihutang OR Da-Chai-Hu-Tang OR Dachaihu Decoction)

Preliminary search date: February 1, 2023

Number of retrieved articles: 1

12、British Library Inside (https://explore.bl.uk/primo_library/libweb/action/search.do?vid1/4BLVU1)

(Pancreatitis OR Acute Pancreatitis OR Acute Edematous Pancreatitis OR Acute Necrotizing Pancreatitis) AND (Dachaihutang OR Da-Chai-Hu-Tang OR Dachaihu Decoction)

Preliminary search date: February 1, 2023

Number of retrieved articles:5

13、ProQuest Dissertations & Theses Global (https://www.proquest.com/index)

(Pancreatitis OR (Acute Pancreatitis) OR (Acute Edematous Pancreatitis) OR (Acute Necrotizing Pancreatitis)) AND (Dachaihutang OR Da-Chai-Hu-Tang OR (Dachaihu Decoction))

Preliminary search date: February 1, 2023

Number of retrieved articles: 10

14、BIOSIS preview (http://gateway.ovid.com/autologin.html)

((Pancreatitis or Acute Pancreatitis or Acute Edematous Pancreatitis or Acute Necrotizing Pancreatitis) and (Dachaihutang or Da-Chai-Hu-Tang or Dachaihu Decoction))

Preliminary search date: February 4, 2023

Number of retrieved articles: 1
