# Supplementary material for: Efficacy and safety of Dachaihu Decoction for acute pancreatitis: Protocol for a systematic review and meta-analysis
Source: PLoS One. 2023 May 18;18(5):e0285661. doi: 10.1371/journal.pone.0285661 (PMC10194870; doi:10.1371/journal.pone.0285661)
Supplement: Attachment — Submitted filename: Response to Reviewers.docx [file pone.0285661.s005.docx]

**Response to Reviwers**

**Dear editor and reviewers:**

We are very grateful for your efforts to deal with our manuscript, and also very grateful for the professional and pertinent comments put forward by the two reviewers. We have revised the corresponding part according to the suggestions of the two reviewers. In this reply, we responded to the comments of the editor and reviewers one by one.

**ACADEMIC EDITOR:** Please check the comments of all reviewers and avoid excessive acronyms in the abstract and throughout the manuscript.

We have carefully checked the comments of the reviewers and revised the corresponding contents in the manuscript. In addition, we also reduced some unnecessary acronyms, such as IPN, ACS, SAP, etc.

**Reviewer #1:** This protocol for systematic review aimed to assess the efficacy and safety of Dachaihu Decoction (DCD) for the treatment of acute pancreatitis (AP). This protocol has been well described and written, and has been registered on PROSPEPRO. However, I have several concerns and questions. The detailed comments are listed below.
**(1) Line 16: Please provide all the ORCIDs of the authors, if possible.**

We have added the ORCID IDs of all authors as requested (**lines 14-21**).
**(2) Line 28-30: If the DCD has been widely administered as an effective treatment, why did a systematic review require? Please point out why this systematic review is necessary, such as there is no sound evidence for this treatment, etc.**

As we reported in the manuscript, DCD was widely used in the treatment of AP, but its safety and effectiveness have not been verified, which limits its application. So we designed this systematic review and meta-analysis (**lines 28-30**).
**(3) Line 32-37, 151: Please also consider electronic datasets (Scopus, CINAHL), grey literature sources (OpenGrey, British Library Inside, ProQuest Dissertations & Theses Global, and BIOSIS preview), and preprint servers, for literature search.**

Thank the reviewer for the comment on the literature retrieval. We agree with the reviewer's suggestion and take electronic databases including Scopus and CINAHL, grey literature sources including OpenGrey, British Library Inside, ProQuest Dissertations&Themes Global, and BIOSIS preview, and preprint servers as our data sources (**lines 32-40, 180, 191-193**).
**(4) Line 55: Please also provide the PDF of your protocol on PROSEPERO as Supplementary Material.**

We have uploaded the protocol registered on the PROSPERO as a supplementary material (**line 57-58**).
**(5) Line 86-86: The DCD has been well described. Please consider provide the amount of these medicinal materials used in the DCD.**

Thanks for the approval and proposal of the reviewer, and we have reported the amount of the medicinal materials used in DCD (**lines 89-92**).

**(6) Line 129: Maybe “has been proved” --> “has shown potential”? The aim of this review is to provide high-quality evidence to “prove” it.**

We are sorry for such a mistake in the manuscript, and we have corrected it to make sure the correctness of the manuscript **(lines 124-125)**.
**(7) Line 133: More description on why the safety of DCD needed to be systematically reviewed. Please provide some disadvantage or adverse effects of DCD in AP treatment to show why this systematic review is needed, maybe the safety of DCD has not been determined?**

Some studies suggested that when DCD was used to treat AP, there might be adverse reactions such as dizziness, peritonitis, bleeding, pancreatic abscess, nausea, and vomiting. We believe that the possible adverse reactions can not be ignored while evaluating its efficacy. Therefore, this study will also explore the safety of DCD (**lines 127-129**).
**(8) Line 153-158: Please provide the specific diagnostic criteria of AP for inclusion instead of citing references.**

We have provided the specific diagnostic criteria of AP in the manuscript according to the requirements of the reviewer (**lines 157-160**).
**(9) Line 178: Chinese Science Journal Database (VIP)?**

I'm sorry that such an inappropriate abbreviation appears in the manuscript. The correct database name is "VIP Database", which has been added to the text (**lines 34; lines 181**).
**(10) Line 219-222: What is the criteria for Cochrane’s Q statistics?**

Heterogeneity will be assessed using the inconsistency index (I2) and Cochrane's Q test. Heterogeneity will be graded as high (I2 > 75%), moderate (between 50% and 75%), or low (I2 < 50%). And the statistical criteria for Cochrane’s Q test are as follows: when P<0.05, there is significant heterogeneity, otherwise, there is no heterogeneity. We have added the above to the manuscript **(lines 227-228)**.
**(11) Line 208-209, 223-234: Please provide the plan for dealing with publication bias in a separated section.**

We have described “Publication Bias” as an independent chapter according to the requirements of the reviewer (**lines 243-244**).
**(12) Figure S1: The PRISMA 2020 checklist has been published. Please applied the diagram in this latest guideline.**

Thanks for the proposal of the reviewer, we have designed the study selection process according to PRISMA 2020 Flow Diagram and uploaded it as a supplementary material (**lines 202, 269**).

**Reviewer #2:** The authors of this study have developed a protocol for meta-analysis to assess the efficacy and safety of Dachaihu Decoction for acute pancreatitis. There are a few concerns abouth this protocol **(1) Types participants: The authors have to specify the etiology of pancreatitis to assess the efficacy. They have to include studies that have administered this decoction either to alcoholic pancreatitis or to idiopathic pancreatitis patients. They cannot generalize the assessment.**

We have specified the disease type according to the reviewer's opinion. Any type of AP, including biliary AP, alcoholic AP, hyperlipidemic AP, mixed AP, and other/idiopathic AP, will be included in our study (**lines 151-152**). This means that we have not limited the etiology of AP, because the clinical symptoms of AP caused by various causes are similar, and the TCM treatment is also the same.
**(2)Types of interventions: The authors need to include studies that have administered a uniform dosage. Otherwise there may be a hetergenity as the authors have mentioned in the discussion. Since this is only a protocol, they can modify to include uniform dosage, route of administration and duration/severity of disease.**

Thanks for the comments of the reviewers, we ignored the consistency of administration route, drug dose, course, and severity of AP in the study. According to the comment of the reviewers, we improved the type of intervention: the administration route, dosage, and course of treatment of the intervention group and the control group were consistent. However, we do not require all the included studies to use the same route of administration, drug dose, and course of treatment, because each TCM researcher may have different clinical thinking and administration habits. If the above causes lead to high heterogeneity between studies, we will conduct a subgroup analysis. (**line 162-163**).

**(3)English is poor, lot of confusion in the subheading types of comparisons. needs to improve.**

In order to improve the quality and readability of the manuscript, we modified the entire text and invited native speakers to modify the sentence and grammar of the manuscript. At the same time, we have submitted the certificate of English editing as a supplementary document. In addition, we have appropriately revised some subheadings in the manuscript (**line 86, 123, 145, 166, 169, 178, 188**).

Yours sincerely,

Xiang Xiao
