# Supplementary material for: Efficacy and safety of Dachaihu Decoction for acute pancreatitis: Protocol for a systematic review and meta-analysis
Source: PLoS One. 2023 May 18;18(5):e0285661. doi: 10.1371/journal.pone.0285661 (PMC10194870; doi:10.1371/journal.pone.0285661)
Supplement: Attachment — Submitted filename: Response to Reviewers.docx [file pone.0285661.s006.docx]

**Response to Reviwers**

**Dear editor and reviewers:**

Thank you very much for your processing and recognition of our manuscript. We have revised the manuscript again according to the opinions of reviewers and editors.

**Journal Requirements:** Please review your reference list to ensure that it is complete and correct. If you have cited papers that have been retracted, please include the rationale for doing so in the manuscript text, or remove these references and replace them with relevant current references. Any changes to the reference list should be mentioned in the rebuttal letter that accompanies your revised manuscript. If you need to cite a retracted article, indicate the article’s retracted status in the References list and also include a citation and full reference for the retraction notice.

All references have been checked in this revision and we ensure their completeness and accuracy. In addition, we determined that no retracted or amended papers were cited in the manuscript. In this revision, we have added a new reference according to the reviewer's opinion (41.	Zorzela L, Loke YK, Ioannidis JP, et al. PRISMA harms checklist: improving harms reporting in systematic reviews. BMJ. 2016 Feb 1;352:i157. doi: 10.1136/bmj.i157. Erratum in: BMJ. 2016;353:i2229. PMID: 26830668).

**Reviewer #1:** This protocol for systematic review aimed to assess the efficacy and safety of Dachaihu Decoction (DCD) for the treatment of acute pancreatitis (AP). I compared the two versions of manuscript and studied the response letter carefully. I think the authors have revised manuscript well, and the current version is almost suitable for publication. I have only two minor comments.

Thank you very much for your recognition of our work. In addition, for the two comments you put forward in this review, we have revised accordingly and replied one by one below.
**(1) The author decided to include electronic datasets (Scopus, CINAHL), grey literature sources (OpenGrey, British Library Inside, ProQuest Dissertations & Theses Global, and BIOSIS preview) as data sources. Please provide the detailed search strategy for other databases in the Appendix S3. Please also add the preliminary search date and number of retrieved articles in the preliminary search in Appendix S3.**

We are very grateful for the reviewer's comment, which will help to improve the credibility and repeatability of our research. We have provided detailed search strategies for all databases in Appendix S3, indicating the date of the initial search and the number of documents retrieved. It is worth mentioning that the deadline for our pre-search is February 4, 2023, so we have extended the deadline for the official search in the protocol (**Line 39, 183**). As described in the manuscript, the official search deadline was set for May 31, 2023.
**(2) Line 28-30: The author declared that this systematic review is necessary because the safety and effectiveness of DCD in AP have not been verified. Please consider to use the PRISMA harms checklist to improve the harms reporting in your systematic review (BMJ. 2016 Feb 1;352:i157. doi: 10.1136/bmj.i157).**

We have to admit that this is a very meaningful proposal. We accepted the recommendation and stated in the manuscript that this systematic review and meta-analysis will be performed and reported under the PRISMA harms checklist (Line 144-145).

**Reviewer #2:** The authors have answered my comments sufficiently. English is also improved, hence can be accepted.

Thank you very much for your recognition. Of course, we are even more grateful for your suggestions in the first review, which is of vital significance to the smooth development of our research.

Yours sincerely,

Xiang Xiao
